# Identification and characterisation of the haemozoin of *Haemonchus contortus*

**DOI:** 10.1186/s13071-023-05714-3

**Published:** 2023-03-06

**Authors:** Lu Liu, Zongshan Zhang, Hui Liu, Shengnan Zhu, Taoxun Zhou, Chunqun Wang, Min Hu

**Affiliations:** State Key Laboratory of Agricultural Microbiology, College of Veterinary Medicine, Huazhong Agricultural University, Wuhan, 430070 China

**Keywords:** *Haemonchus contortus*, Haemoglobin, Haem, Haemozoin, Lipid

## Abstract

**Background:**

Most haematophagous organisms constantly suck the host’s haemoglobin, which produces toxic free haem. This toxic haem aggregation into the nontoxic crystallisation complex known as haemozoin represents one of the most important detoxification pathways in living organisms, but very little is known about the features of haemozoin in parasitic nematodes. Here, we identified and characterised the haemozoin of an economically significant blood-sucking nematode, *Haemonchus contortus*.

**Methods:**

Using electron microscopy, spectrophotometry analyses and biochemical approaches, haemozoin crystallisation was identified and characterised in parasitic fourth-stage larvae (L4s) and/or adult worms as well as L4s of in vitro culture.

**Results:**

The haemozoin was formed in intestinal lipid droplets of the parasitic L4s and adult worms. The characterisation of the haemozoin showed regularly spherical structures and had a 400-nm absorption peak. Furthermore, the haemozoin in in vitro cultured L4s was associated with the culture time and concentration of red blood cells added into the medium, and its formation could be inhibited by chloroquine-derived drugs.

**Conclusions:**

This work provides detailed insight into the haemozoin formation of *H. contortus* and should have important implications for developing novel therapeutic targets against this parasite or related haematophagous organisms.

**Graphical abstract:**

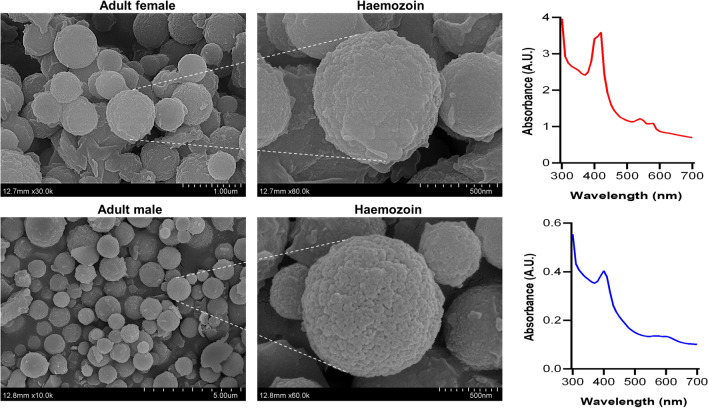

## Background

Blood sucking is a common feature that is shared by various organisms from protozoa to mammals [1, 2]. These haematophagous organisms usually digest large quantities of host haemoglobin to acquire essential nutrients for their development [3]. During the digestion process of haemoglobin, it is degraded into many byproducts including amino acids, peptides and haem. Haem is an essential prosthetic group that composes an integral molecule in most organisms. In fact, most blood-sucking organisms are absent in the de novo synthesis of endogenous haem and have to acquire it through their host haemoglobin [2, 4]. However, haem in a free state is highly toxic because of its nature as a pro-oxidant, which constitutes a major threat to living organisms, including inducing the formation of free radicals, interfering with phospholipid bilayer stability as well as accelerating cell cytolysis [5]. Thus, haematophagous organisms can make the toxic haem become nontoxic; these processes are essential to their physiologies.

Indeed, many efficient detoxification paths of haem have evolved in such haematophagous organisms. Evidence from hookworms has shown a haem catabolism pathway involving many glutathione-*S*-transferase proteins [6]. Similarly, this pathway has been described in malaria parasites *Plasmodium* spp. and other blood-feeding parasites [7–10]. In addition, there is a widely described pathway of haem detoxification that involves free haem aggregation into the crystallisation complex known as haemozoin [11–14]. This formed haemozoin has a dark-brown ferric pigment that has been characterised in both *Plasmodium* spp. and blood flukes *Schistosoma* spp. [15, 16], representing one of the most important detoxification pathways. Given the formation of haemozoin in such related parasites, we hypothesised it is a possible presence in the haematophagous *Haemonchus contortus* (barber pole worm)—one of the most pathogenic nematodes in ruminant animals.

The life cycle of *H. contortus* consists of free-living egg to third-stage larva (L3) stage and parasitic fourth-stage larva (L4) to adult stage [17]. The latter parasitic stages of *H. contortus* have to suck the host blood to provide an indispensable source of nutrition for their growth and reproduction [18]. It is estimated that individual adult worms are able to ingest 30–50 μl blood per day [19, 20]. Although the parasite needs to degrade large amounts of haemoglobin, no information is known about the features of haemozoin formation in this parasite. The aim of the present study was to characterise the haemozoin of *H. contortus* using electron microscopy analyses and biochemical approaches. This work provides the first detailed insight into the haemozoin formation of *H. contortus* and will lay the foundation for developing novel therapeutic targets against this parasite or related haematophagous organisms.

## Methods

### Ethics statement

The animal experiment was approved by the Experimental Animals Ethics Committee of Huazhong Agricultural University (HZAUGO-2019-008). The care and maintenance of all animals were in accordance with the protocols and guidelines directed by the committee.

### Parasite materials

Different developmental stages of *H. contortus* (Haecon-5 strain) were collected, processed and stored based on well-established protocols [21]. Specifically, infective L3s were collected from coproculture at 25 °C [22] and maintained at 15 °C. Exsheathed L3s (xL3s) were obtained by incubation with 0.15% (v/v) sodium hypochlorite at 37 °C for 10 min [23]. L4s and adult worms were identified and collected from the infected goat’s abomasa at days 7 and 28 post-infection, respectively [17]. To consume the host haemoglobin, collected worms were starved for 8 h in phosphate-buffered saline (PBS).

### Light microscope analysis of haemozoin-like pigements

The collected parasitic L4s and adult *H. contortus* were thoroughly washed using sterile PBS solution. The samples were anesthetised by 1% levamisole and then the haemozoin-like pigement was observed by a light microscope (Zeiss Primovert iLED, Gttingen, Germany). To identify the ferric iron in the haemozoin, these L4s and adult worms were stained by a ferric iron stain kit (Solarbio, Beijing, China) according to the manufacturer’s instructions. Briefly, these parasites were fixed with 4% paraformaldehyde for 24 h and stained for 24 h. After the worms were washed and dehydrated, they were carefully transferred to a glass slide and observed using Zeiss light microscopy.

### Transmission electron microscopy (TEM) analysis

To observe the haemozoin distribution in *H. contortus*, TEM analysis was performed as previously described [24]. In brief, fresh L4s and adult worms were fixed in 0.1 M cacodylate buffer (pH 7.4) with 2.5% glutaraldehyde and 4% formaldehyde. Fixed samples were dehydrated, embedded and then stained with uranyl acetate and lead citrate. Final samples were observed by TEM analysis (Hitachi H-7650, Tokyo, Japan).

### Field emission scanning electron microscopy (FESEM) analysis

Haemozoin was extracted from adult *H. contortus* using a well-established method [25]. In brief, 100 adult worms were homogenised (30 min, 4 °C) and ultrasonicated (5 min, 4 °C). The homogenate was centrifuged at 12,000*g* for 10 min. The collected supernatant was centrifuged at 12,000*g* for 15 min. The pellet was washed and incubated with 1 mg/ml proteinase K (Yuanye, Shanghai, China) at 37 °C overnight. After the sample was washed again and froze dried, the samples were transferred into a silicon chip coated with gold (10 min), followed by observation using a SU8010 FESEM (Techcomp, Shanghai, China) at an accelerating voltage of 5.0 kV.

### Spectrophotometry analysis

The haemozoin was qualitatively analysed as described previously [26]. Briefly, haemozoin extracts were dissolved in 0.1 M NaOH solution and the sample (200 μl) was added to the 96-well plates. The wavelength of haemozoin was determined at 300–700 nm or 400 nm using a multi-mode plate reader (BioTek Cytation 5, Winooski, VT, USA).

### Protein and lipid extraction from adult worms

Protein and lipid samples were isolated using well-established methods [27]. Briefly, female and male worms were respectively homogenised in PBS buffer (pH 7.4) with 1 M proteinase K inhibitor. The homogenate was centrifuged (10,000*g* for 10 min) and the supernatant collected. Protein concentration was assessed by a bicinchoninic acid assay (BCA) kit (Beyotime Biotechnology, Shanghai, China).

For lipid extraction, 1 ml *H. contortus* homogenate (female or male) was fully mixed in an extract liquor with 4 ml chloroform and 2 ml methanol. The sample was vigorously shaken (5 min) and subsequently centrifuged (1000*g* for 10 min). The upper aqueous phase was discarded and the lower organic phase dried in the N_2_ stream. The lipids extracted were gravimetrically determined by an analytical balance. The dried lipids were solubilised in 100% chloroform and stored at − 80 °C until used.

### Haem aggregation assay

To determine whether proteins and lipids promote the formation of β-haematin, a haem aggregation assay was carried out [27]. In brief, 100 mM bovine-derived haem (Beyotime, Shanghai, China) was dissolved in 0.5 M sodium acetate (pH 4.8) with 20 mg protein and 80 μg lipid, respectively. The reaction was incubated at 37 °C for 24 h prior to centrifuge at 12,000*g* for 10 min. The final pellet was solubilised in 0.1 M NaOH buffer, and the wavelength of β-haematin was determined at 400 nm based on the above spectrophotometry analysis.

### L4s culture in vitro

L4s were cultured in vitro based on a previous report with some modifications [21]; 100 L3s were cultured in 24-well plates with Luria Bertani and NCTC-109 medium and supplemented with 20% foetal bovine serum (FBS) and 100 IU/ml penicillin, 100 μg/ml streptomycin and 25 μg/ml amphotericin (Antibiotic-Antimycotic, Sigma-Aldrich, St. Louis, MO, USA). To promote xL3s into L4s, the larvae were incubated in a 20% CO_2_ incubator at 39 ℃ for 7 days. Subsequently, 12.5% goat's deflbrinated blood (DFB) was added to the medium.

At 48 h cocultivation, haemozoin-like pigments were observed in L4s cultured with/without DFB and spectrophotometrically determined at 300–700 nm. Also, the wavelength of haemozoin was determined at 400 nm after 24 h, 48 h and 72 h of cocultivation. To determine the effect of red blood cells (RBC) concentration, we isolated the RBC from the goat FBS. After adding the 10^7^, 10^8^ and 10^9^ RBC, the wavelength of haemozoin was determined spectrophotometrically at 72 h.

### Quinolone-drug inhibition analysis

To assess the inhibition of quinolone drugs, different concentrations (0 nM, 1 nM, 10 nM and 100 nM) of mefloquine (Macklin, Shanghai, China) and quinine (Macklin, Shanghai, China) were added to the L4s (*n* = 100) cultured medium containing 12.5% DFB. The wavelength of haemozoin was spectrophotometrically determined at 72 h of cocultivation.

### Statistical analysis

Statistical analysis was conducted using GraphPad Prism 8.0 software (GraphPad Software, San Diego, CA, USA). One-way ANOVA was applied to compare the statistical differences between groups. *p < 0.033, **p < 0.002 and ***p < 0.001 indicate the degree of significance

## Results

### Haemozoin-like pigments formed in *Haemonchus contortus* intestine

**Fig. 1:**
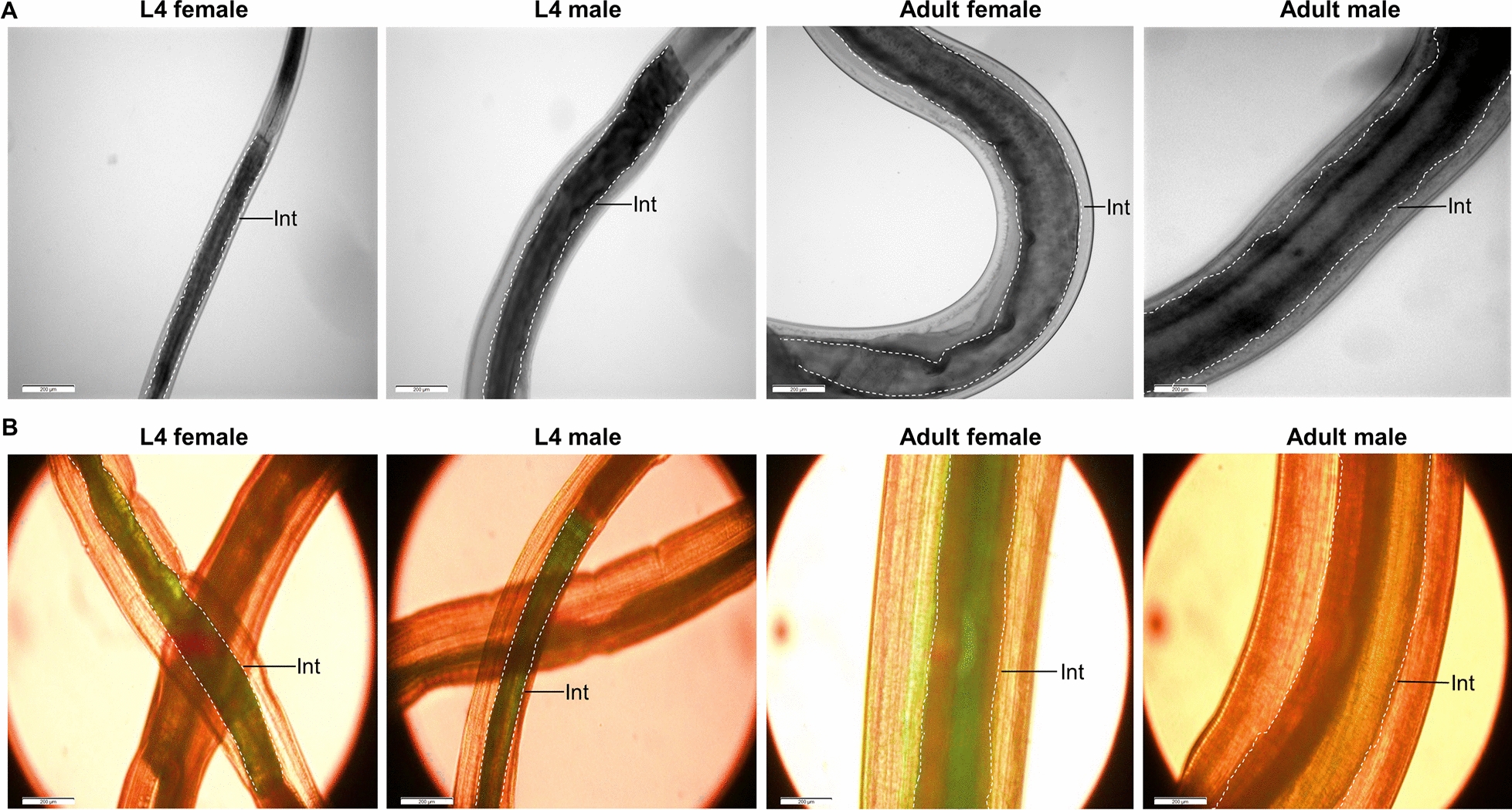
Light microscope analysis of haemozoin-like pigments in parasitic-stage *Haemonchus contortus*. Parasitic fourth-stage larvae (L4s) and adult worms were collected from the infected goat’s abomasa at days 7 and 28 post-infection, respectively. **A** Representative light microscope images showing the haemozoin-like pigments in L4s and adult worms. **B** Representative light microscope images showing ferric iron stain (green) in L4s and adult worms. The intestinal tissues are outlined by dotted lines. Three independent replicates were performed with similar results. Scale bar = 200 μm. Int, intestine

Light microscope analysis showed haemozoin-like pigments in the intestines of the parasitic L4s and adult *H. contortus* (Fig. 1A). Because the haemozoin is a ferric ion polymer [28], the L4s and adult worms were stained by a ferric ion stain. The integral intestines of L4s and adult worms showed obvious blue green and the deepest colour was observed in the foregut (Fig. 1B), consistent with the formation position of haemozoin-like pigments, suggesting that the dark black pigments could be haemozoin.

### Haemozoin located in the intestinal lipid droplets

**Fig. 2:**
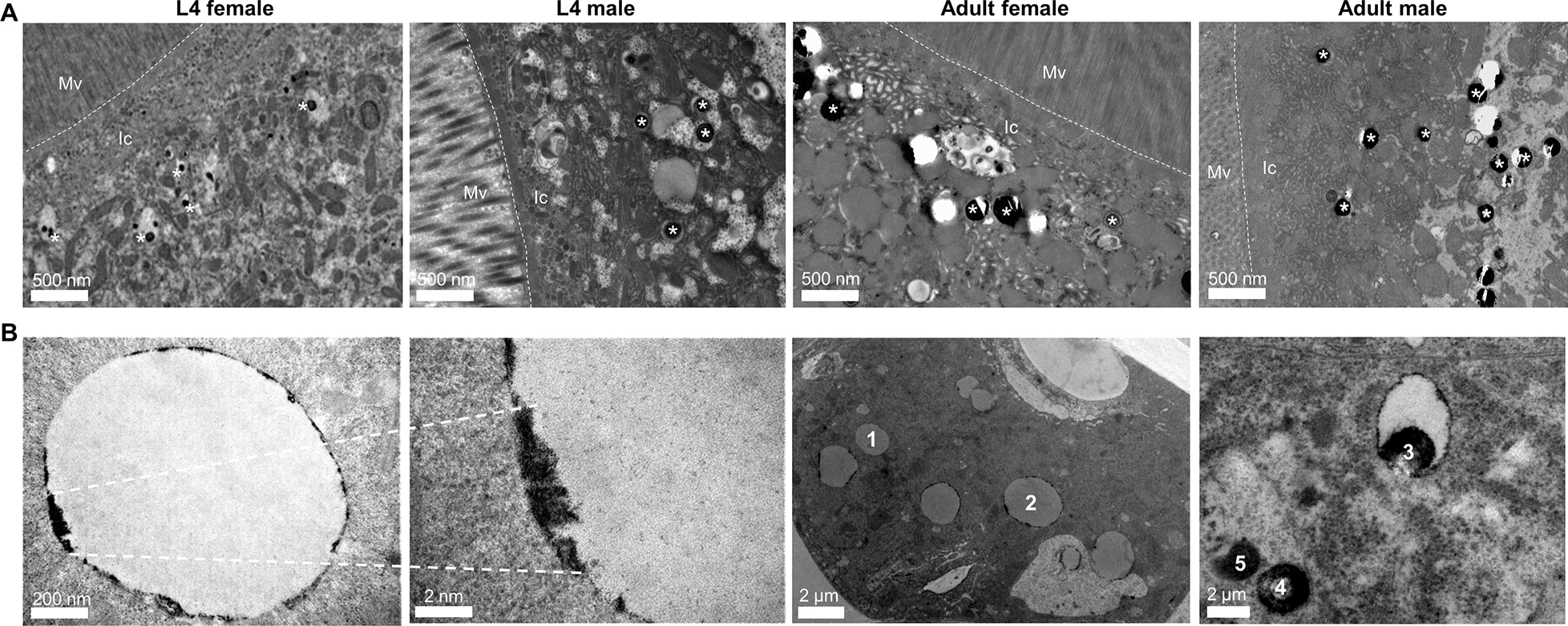
Transmission electron microscopy (TEM) analysis of haemozoin in lipid droplets of *Haemonchus contortus*. Parasitic fourth-stage larvae (L4s) and adult worms were collected from the infected goat’s abomasa at days 7 and 28 post-infection, respectively. **A** Representative TEM images displaying haemozoin in the lipid droplets (LDs) in L4s and adult worms. Aggregated haemozoin is marked with asterisks*.* The intestinal inner walls were outlined by dotted lines. **B** Representative images showing haemozoin associated with LD surface and some different stages of haemozoin formation; the putative sequence of haemozoin formation is indicated by numbers (1: no crystal to 5: almost complete crystal). Three experimental replicates were performed with similar results. Scale bar = 2 nm, 200 nm, 500 nm and 2 μm. Mv, microvilli; Ic, intestinal cytoplasm

**Fig. 3:**
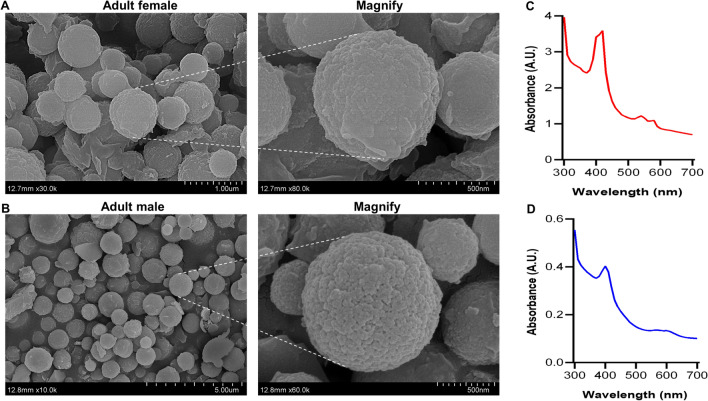
Field emission scanning electron microscopy (FESEM) and spectral scanning analyses of haemozoin isolated from adult female and male worms. Representative FESEM images exhibiting the appearance characteristics of haemozoin isolated from adult female (**A**) and male (**B**) worms. The wavelength of haemozoin from the adult female (**C**) and male (**D**) worms was spectrophotometrically determined at 300–700 nm

**Fig. 4:**
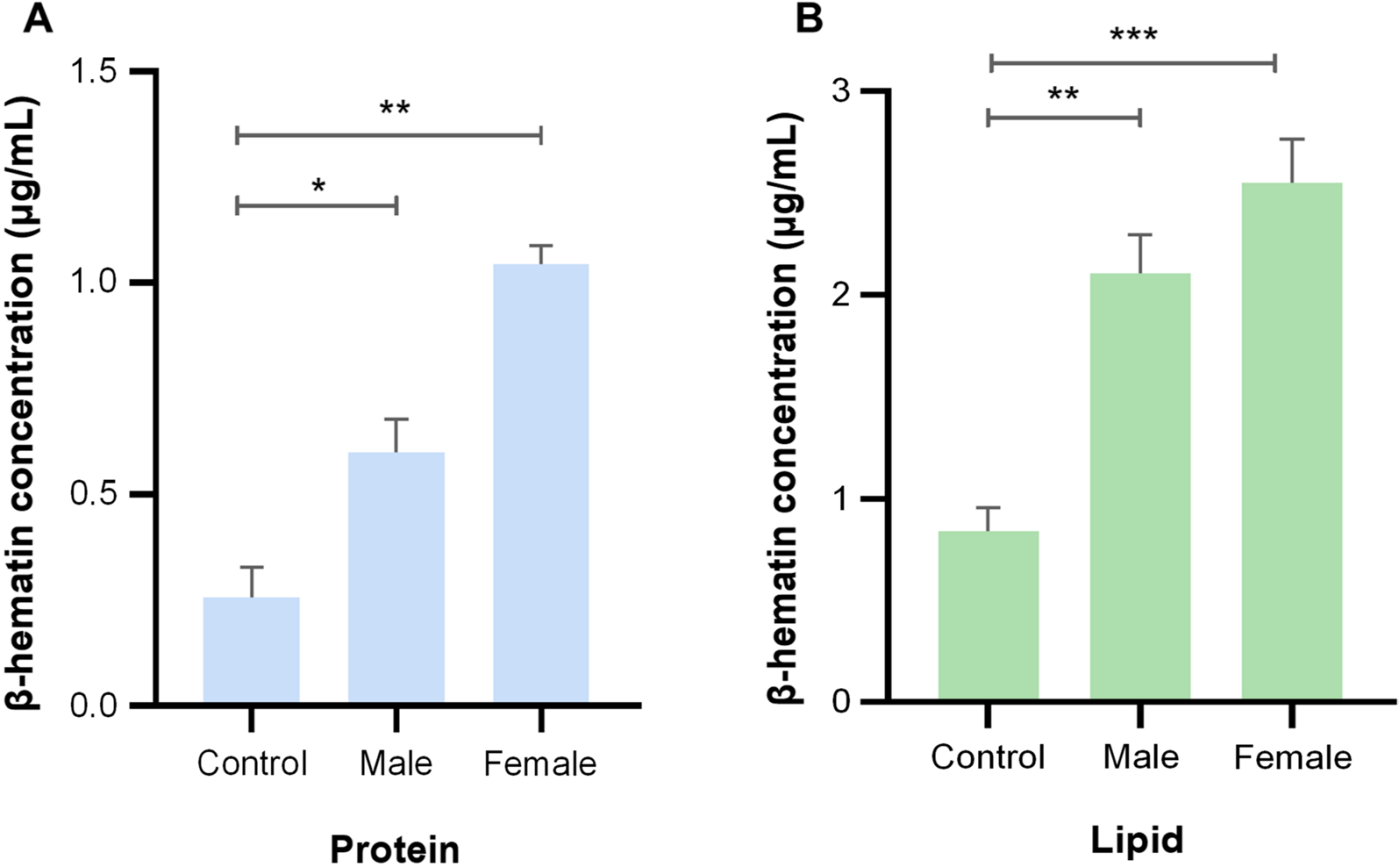
Proteins and lipids isolated from adult worms promote the formation of β-haematin. The bovine-derived haem (100 mM) was dissolved in sodium acetate with 20 mg protein (**A**) and 80 μg lipid (**B**) of adult female and male worms, respectively. After the reaction was incubated and centrifuged, the wavelength of haemozoin was determined at 400 nm based on a spectrophotometry analysis. Data were from three independent replicates. Statistical significance was determined by one-way ANOVA and indicated by asterisks, *p < 0.033, **p < 0.002 and ***p < 0.001

To discern haemozoin distribution patterns in *H. contortus* intestine, we observed the lipid droplets using TEM analysis. We showed haemozoin forming in lipid droplets of intestinal cytoplasm of parasitic L4s and adult worms (Fig. 2A). Specifically, the different stages of haemozoin-like crystallisation were closely associated with lipid droplets. Early crystallisation was found in the surface of lipid droplets, and these small crystals could gradually form larger crystals toward the core of lipid droplets (Fig. 2B). To determine the characteristics of these crystals, we isolated the haemozoin from female and male *H. contortus* and performed the morphological observation. The results revealed that all crystals were roughly spherically shaped with 5–500-nm diameter (Fig. 3A, B) and had a 400-nm absorption peak (Fig. 3C, D). Extending this work, we demonstrated that proteins and lipids from adult worms could promote β-haematin formation in vitro (Fig. 4A, B).

### Haemozoin could be form in L4s in vitro

**Fig. 5:**
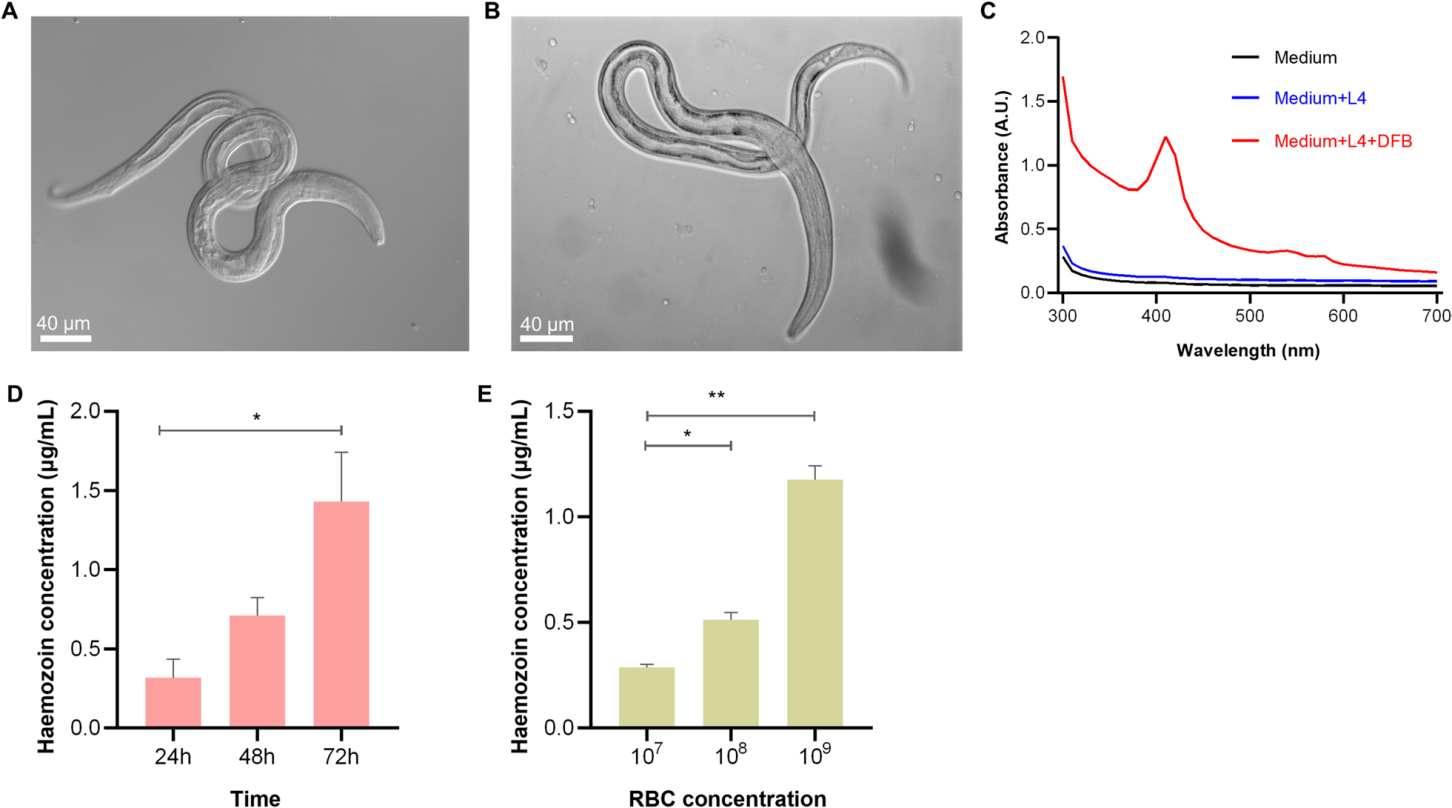
Haemozoin-like pigments form in L4s of in vitro culture. Based on previous culture conditions of L4s in vitro, additional 12.5% deflbrinated blood (DFB) from goat was added. **A, B** At 48 h of cocultivation, haemozoin-like pigments were microscopically observed in L4s cultured with/without DFB. Scale bar = 40 μm. **C** Wavelengths of haemozoin were determined (300–700 nm) in L4s cultured in normal medium and with/without DFB.** D** After 24 h, 48 h and 72 h of cocultivation, the wavelength of haemozoin was spectrophotometrically determined at 400 nm. **E** Based on normal culture conditions of L4s in vitro, an additional 10^7^, 10^8^ and 10^9^ red blood cells (RBC) were supplemented. The wavelength of haemozoin was determined at 72 h of cocultivation. Data were from three independent replicates. Statistical significance was determined by one-way ANOVA and is indicated by asterisks, *p < 0.033 and **p < 0.002

Having shown that the haemozoin can form in the parasitic stages of *H. contortus*, we assessed whether these crystals could be formed in L4s in vitro. As expected, haemozoin-like pigments were observed in the L4s cultured with the presence of the goat DFB, whereas these pigments were not shown in the L4s lacking DFB medium (Fig. 5A, B). In the former group of L4s, a 400-nm haemozoin-related absorption peak was identified (Fig. 5C). To assess the effect of blood on haem crystallisation, we cultured L4s in 12.5% DFB medium for 24 h, 48 h and 72 h and showed 72 h of coculture had a higher haemozoin concentration compared with 24 h and 48 h (Fig. 5D). When culturing L4s in RBC medium with a concentration gradient, the production of haemozoin was concentration-dependent (Fig. 5E). These data suggest that haem crystals could be formed in L4s in vitro, which could represent a key detoxification pathway.

### Haemozoin could be inhibited by chloroquine drugs

**Fig. 6:**
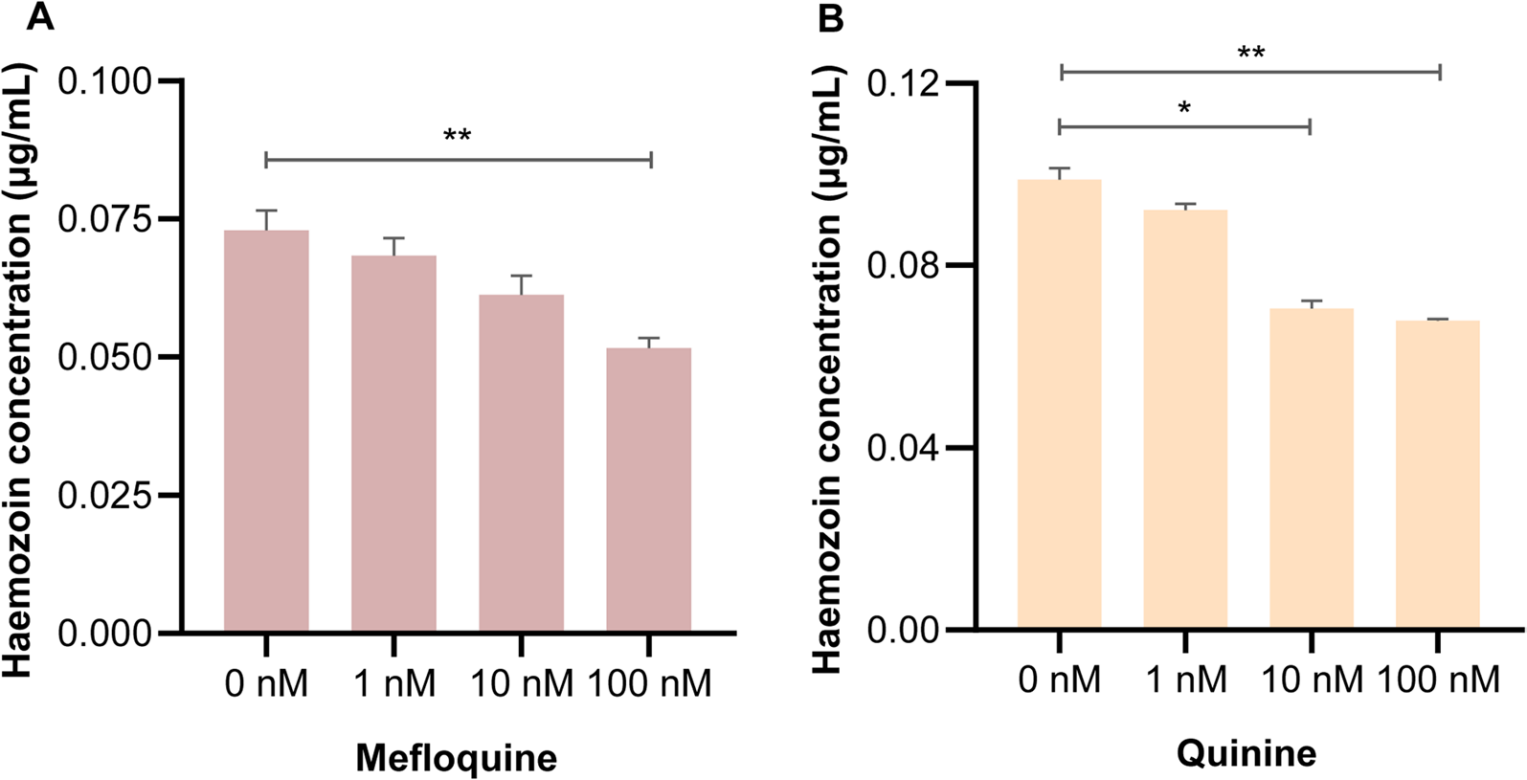
Chloroquine drugs inhibit the formation of haemozoin. **A, B** L4s culture medium containing 12.5% deflbrinated blood (DFB) was supplemented with different concentrations (0 nM, 1 nM, 10 nM and 100 nM) of mefloquine and quinine. The wavelength of haemozoin was determined at 72 h of cocultivation. Data were from three independent replicates. Statistical significance was determined by one-way ANOVA and indicated by asterisks, *p < 0.033 and **p < 0.002

Given the importance of haemozoin in parasite growth, we verified that mefloquine (100 nM) and quinine (10 nM and 100 nM) could strongly inhibit haemozoin synthesis when L4s were fed with blood supplemented with different concentrations of chloroquine-derived drugs in vitro (Fig. 6A, B), consistent with a previous study [29], suggesting the chloroquine-derived constituents could be potential drugs for controlling haematophagous parasites.

## Discussion

Many haematophagous parasites have to suck the host’s blood because many blood components provide an indispensable source of nutrition for their growth and survival [26, 30, 31]. Haemoglobin, as a major blood component, is a haem-containing protein [32, 33] and is important to accomplish the transition of parasite life and reproduction of adult worms [34–36]. However, during the proteolytic digestion of this haemoglobin, free haem constitutes a major threat to parasite development because of its toxic action including induction of oxygen-derived free radicals and lysis of normal cells [37, 38]. After a long-term study of how the toxic haems become nontoxic, it has been suggested that haem in haemozoin crystallisation represents a dominant pathway of haem detoxification in blood-feeding parasites [39].

Here, we investigated the features of haemozoin derived from the blood-sucking *H. contortus*—one of the most important parasitic nematodes of livestock. Using electron microscopy analyses and biochemical methods, we showed that haemozoin was formed in intestinal lipid droplets of parasitic L4s and adult worms, and its appearance was regularly spherically shaped with a 400-nm absorption peak. We also revealed that the formation of haemozoin was associated with culture time and concentration of red blood cells in in vitro cultured L4s and could be inhibited by chloroquine-derived drugs.

The parasitic L4s and adult worms need to ingest a large amount of haemoglobin in their intestine to maintain their normal development and reproduction [40], so it is no surprise that we showed the intestine as a major organ forming haemozoin-like pigments. Recently, the mechanism of haem acquisition of *H. contortus* has also shown that the intestine is an important organ where haem-responsive gene-1 protein (HRG-1) can facilitate haem uptake and utilisation [41]. We observed that the haemozoins in lipid droplets were located in intestinal cytoplasm, which is in line with hookworm *Nippostrongylus brasiliensis* [6], but different from helminth *Schistosoma mansoni* and insect *Rhodnius prolixus* [42]. Haemozoin of the latter two species is present only in the intestinal lumen [43, 44], although lipid droplets are also observed in the intestinal cytoplasm. So far, the mechanism of haemozoin formation is still not fully understood. Our results revealed that lipids and proteins could play a key role in catalysing haemozoin formation and could be initiated at the hydrophilic-hydrophobic interface, but which types of proteins and lipids promote haemozoin crystallisation remain to be further studied.

Morphological analysis showed haemozoin of *H. contortus* was very regular, but the size was not consistent. The haemozoin of *H. contortus* displayed a roundly spherical structure, and these spherical crystallites looked more standard compared to the crystallite structure of *S. mansoni* [42]. This could be why haemozoin is in an orderly arrangement in lipid droplets. Intriguingly, the haemozoin in *H.contortus* was obviously different from other haematophagous organisms, such as *Plasmodium* species and *R. prolixus*, showing the rod-shaped structures extend in different directions [25]. In regarding the size of haemozoin, many organisms including *S. mansoni* and *R. prolixus* have shown inconsistency with *H. contortus*; even many crystals in *S. mansoni* can reach the micron scale [42]. Therefore, although haemozoin molecules in different species all consist of free haem, their formation characteristics may be slightly different between different blood-sucking species.

During the culture process of L4s in vitro, haemozoin was only observed in the presence of a DFB medium and its formation could be affected by the culture time and concentration of red blood cells in the medium. In fact, most blood-sucking parasites still do not supplement haemoglobin or blood in the culture medium, which may be an important reason for the failure of in vitro culture, because the in vitro culture medium without blood could lack the source of exogenous haem to support their growth. In support of this opinion, a study of *S. mansoni* has shown that supplementing blood and many substances in the culture medium could make adult *S. mansoni* produce hatching eggs [35]. In turn, blocking the formation of haemozoin may inhibit the development of parasites. We verified that chloroquine-derived drugs could strongly inhibit *H. contortus* haemozoin synthesis. Such chloroquine-derived drugs have been proposed to target haemozoin formation to inhibit many other parasites [45–47], but the optimal inhibitory concentration and whether these chloroquine drugs can be used as a new drug still needs to be investigated further.

## Conclusion

In the present study, we identified and characterised *H. contortus* haemozoin. This haemozoin was regularly formed in intestinal lipid droplets of parasitic L4s and adult worms. We revealed that the haemozoin in in vitro cultured L4s was associated with the culture time and concentration of red blood cells, and its formation could be inhibited by chloroquine-derived drugs. Our work, therefore, provides insight into haemozoin formation in *H. contortus* and will facilitate the development of novel therapeutic targets against related haematophagous parasites.

## Data Availability

Not applicable.

## Abbreviations

PBS: Phosphate-buffered saline
BCA: Bicinchoninic acid assay
L3s: Third-stage larvae
xL3s: Exsheathed L3s
L4s: Fourth-stage larvae
LD: Lipid droplet
TEM: Transmission electron microscopy
FESCM: Field emission scanning electron microscopy
FBS: Foetal bovial serum
DFB: Defibrinated blood
RBC: Red blood cells
HRG-1: Haem-responsive gene-1 protein
Int: Intestine
Mv: Microvilli
Ic: Intestinal cytoplasm
